# Fungal Pneumonia in The Immunocompetent Host: A Possible Statistical Connection Between Allergic Fungal Sinusitis with Polyposis and Recurrent Pulmonary Infection Detected by Gastroesophageal Reflux Disease Scintigraphy

**DOI:** 10.4274/mirt.galenos.2020.32154

**Published:** 2020-04-29

**Authors:** Leticia Burton, Karl Baumgart, Daniel Novakovic, John Beattie, David Joffe, Gregory Falk, Hans Van der Wall

**Affiliations:** 1 University of Notre Dame, CNI Molecular Imaging, Sydney, Australia; 2 North Shore Medical Centre, Sydney, Australia; 3 University of Sydney, Sydney, Australia; 4 Ryde Medical Centre, Sydney, Australia; 5 Royal North Shore Hospital, Sydney, Australia

**Keywords:** Allergic, fungal, rhinosinusitis, pneumonia, reflux, scintigraphy

## Abstract

**Objectives:**

Fungal pneumonia in the immune competent host is a rarity with few reported cases in the literature. We present a series of 7 cases of recurrent fungal pneumonia in association with allergic fungal rhinosinusitis and gastroesophageal reflux disease (GERD). We hypothesised that recurrent infection may have been transported from the infected paranasal sinuses into the lung by GERD as the process was terminated by surgical fundoplication in 2 of these patients.

**Methods:**

Patients were recruited into the study if they were immune competent and had recurrent fungal pneumonia and GERD. Allergic fungal rhinosinusitis was proven by biopsy. GERD was investigated by a scintigraphic test that assessed local oesophageal disease, lung aspiration and head and neck involvement with a hybrid gamma camera and X-ray computed tomography.

**Results:**

All patients were shown to have GERD with 5/7 showing paranasal sinus contamination and 7/7 showing laryngopharyngeal involvement and 6/7 lung aspiration. One patient had characteristics strongly predictive of aspiration. Fundoplication led to cessation of fungal lung infection in two patients.

**Conclusion:**

Recurrent fungal pneumonia in the immune competent host should raise the possibility of re-infection from the paranasal sinuses, especially in patients with GERD.

## Abstract (tr)

**Amaç:**

İmmünokompetan hastada fungal pnömoni literatürde az sayıda olguda bildirilmiştir. Bu yazıda alerjik fungal rinosinüzit ve gastroözofageal reflü hastalığı (GÖRH) ile ilişkili tekrarlayan fungal pnömonisi olan 7 olgu sunulmuştur. Bu hastaların 2’sinde cerrahi fundoplikasyon ile tekrarlayan fungal pnömoni atakları sonlandığı için, tekrarlayan enfeksiyonun enfekte paranazal sinüslerden GÖRH ile akciğere taşındığını varsaydık.

**Yöntem:**

Tekrarlayan fungal pnömonisi ve GÖRH olan immünokompetan hastalar çalışmaya alındı. Alerjik fungal rinosinüzitin varlığı biyopsi ile kanıtlandı. GÖRH, lokal özofagus hastalığı, akciğer aspirasyonu ve baş-boyun tutulumunu bir hibrit gamma kamera ve X-ışını bilgisayarlı tomografi ile değerlendiren bir sintigrafik test ile araştırıldı

**Bulgular:**

Tüm hastalarda GÖRH olduğu gösterildi. Yedi hastanın 5’inde paranazal sinüs kontaminasyonu, tamamında laringofaringeal tutulum ve 6’sında akciğer aspirasyonu gösterildi. Bir hastada aspirasyonun olduğunu güçlü bir şekilde öngören özellikler vardı. Fundoplikasyon iki hastada fungal akciğer enfeksiyonunun kesilmesine yol açtı.

**Sonuç:**

İmmünokompetan hastalarda tekrarlayan fungal pnömoni, özellikle GÖRH olan hastalarda paranazal sinüslerden re-enfeksiyon olasılığını düşündürmelidir.

## Introduction

The Aspergillus species of fungus is widespread and generally acquired by inhalation of airborne spores. The immunocompetence of the host is a critical factor in the establishment of invasive infection. More rarely, infection may occur in the immunocompetent host, as has been described by a number of authors (1,2,3). Such cases have been described since 1959 (4). These cases include patients with chronic fungal infections of the maxillary sinuses, mediastinum, lymph nodes and direct pulmonary involvement. The cohort in which the biggest of these series has been described (3) have consisted relatively young men and women. In that series 9 of 18 patients had allergic fungal sinusitis with polyposis with a background of chronic rhinosinusitis.

This paper presents 7 patients with recurrent pulmonary infections who are distinguished from most previous reports in terms of a connection between recurrent fungal pneumonia and allergic fungal sinusitis with polyposis. Several of these patients had undergone lobectomy to eradicate the primary infection of the lungs with subsequent recurrence elsewhere in the lungs. All patients gave a history of symptomatic gastroesophageal reflux disease (GERD), and were tested with a scintigraphic reflux study (5,6) to evaluate the presence of disease within the oesophagus, paranasal sinuses and the possibility of aspiration of refluxate into the lungs. The findings led to exploration of a possible connection between these conditions and surgical intervention, supporting the conclusions.

Based on this case series, we hypothesised that there might be a connection between severe GERD with aspiration into the lungs, recurrent pulmonary fungal infections and allergic fungal sinusitis with polyposis.

## Materials and Methods

### Patient Group

Consecutive patients with fungal pneumonia were referred to a single Nuclear Medicine practice as part of a large research study to evaluate extra-oesophageal manifestations of GERD over a period of 3 years. All patients had established GERD on the basis of 24-hour pH, manometry and impedance monitoring and most had undergone upper gastrointestinal endoscopy and ear, nose and throat assessment with laryngoscopy. The presence of allergic fungal sinusitis with polyposis had been confirmed by biopsy of tissue from the paranasal sinuses, although culture had been unsuccessful in 3 of 7 cases. There was no evidence of invasion or mycetoma formation in these patients. Microscopy of lung tissue obtained by lobectomy or bronchoscopy confirmed semi-invasive fungal disease (7 aspergillus species) in all patients. Immunological testing had confirmed immune competence in all patients. Patients with fungal disease being treated for malignancy or following organ transplantation were excluded from the study.

All patients were on proton pump inhibitor (PPI) therapy at the time of the study and were clinically assessed with the Belafsky Reflux symptom index score (7).

### Ethical Considerations

A database of patients with either proven or suspected GERD/Laryngopharyngeral reflux (approved by the Institutional Ethics Committee of University of Notre Dame 015149S) was maintained prospectively.

### Statistical Analysis

All statistical analysis was performed on the Statistical Package for the Social Sciences (SPPS Version 24, IBM, New York, USA).

### Scintigraphic Reflux Study

Patients were fasted for 12 hours and medications ceased for the 24-hours prior to the test. While upright, patients were positioned in front of a Hawkeye 4 gamma camera (General Electric, Milwaukee, USA) with markers on the mandible and stomach to ensure regions of interest were in the field of view of the camera. Patients consumed 50-100 mL of water with 60-100 MBq of Technetium Phytate followed by flushing with 50 mL of water to clear the mouth and oesophagus from radioactivity. Dynamic images of the pharynx, oesophagus and stomach were obtained for 2 minutes at 15 secs per frame into a 64x64 matrix while upright. This was followed by a 30-minute dynamic in the supine position. Delayed images were obtained at 2 hours to assess the presence of aspiration of tracer activity into the lungs. Following acquisition of the planar image of the lungs, a single photon emission computed tomography (SPECT) study of the head, neck and lungs was obtained and registered with X-ray computed tomography (CT) of the region. These images were reconstructed, fused and displayed in standard projections. Dynamic images were analysed by time activity curves over the pharynx/laryngopharynx, upper and lower half of the oesophagus and by a background region over the right side of the chest, away from the stomach and oesophagus. Delayed images were analysed by a line profile over the lungs. Time activity curves were graded as showing no GERD, falling, flat or rising curves. Area under the curve and maximal amplitude compared to background were estimated. Liquid gastric emptying half-time was determined from the 30-minute supine acquisition with a single exponential fit to the data.

## Results

Patient data. A total of 7 patients were included in the study with an average age of 62 years (range= 47-74 years). There were 5 females and 2 males. The average length of history of sinusitis was 7 years (range=4-8 years). All had been treated for recurrent biopsy-proven allergic fungal sinusitis with polyposis of the paranasal sinuses and lung infection over a period of approximately 4-5 years. Five of 7 patients were on concurrent therapy for asthma, although lung function testing had shown no reversible component. Three patients had undergone lobectomy of the lungs to eradicate the infection without success. All patients had undergone paranasal sinus surgery with recurrence of infection within weeks of the surgery. Biopsy of tissue from the paranasal sinuses/lungs failed to grow the fungus in media in 3 patients, although microscopy confirmed semi-invasive disease in all 7 patients. Antifungal antibiotics utilised in treatment included erbinafine, fluconazole, itraconazole, voriconazole, posaconazole and griseofulvin.

High resolution CT scanning of the lungs demonstrated pulmonary fibrosis in 3 patients, with bronchiectasis in 2 of these and a further patient with bronchiectasis.

All 7 patients had previously been investigated for GERD with 24-hour pH manometry and impedance that confirmed reflux disease and been commenced on long-term PPI and other therapy (Nexium-5, Somac-3, Losec-1, Ranitidine-4, Tazac-1, Motilium-1). Four patients had no symptoms of heartburn, globus or regurgitation, while 3 gave a history of daily symptoms. The average Belafsky score (7) was 20.0 (range: 0-35.0).

Two of the seven patients who had undergone lobectomy for recurrent fungal pneumonia and shown lung aspiration of refluxate in the scintigraphic studies underwent laparoscopic fundoplication. These patients became disease free in the lungs within 3 months while on antifungal therapy and at follow-up showed reduced parameters of reflux and no further lung aspiration of refluxate.

**Figure 1:**
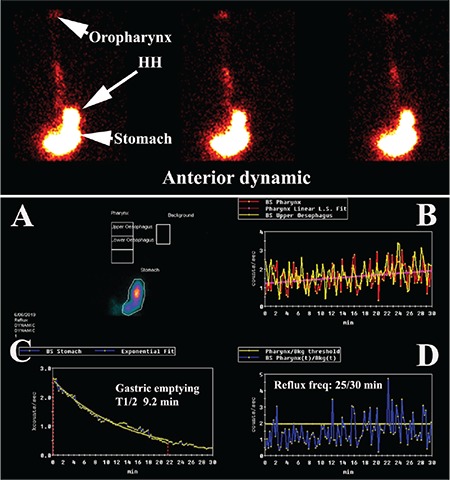
Initial anterior dynamic image with the analysis of the dynamic image in the panel below. Three 15 second frames of the supine dynamic image are shown in the panel above with the oropharynx and stomach labelled. The patient also has a hiatus hernia. The analysis in the panel below shows the regions of interest in A and the time-activity curves for the pharynx/laryngopharynx (red) and osesophagus (yellow) and the curve fitted to the pharynx/laryngopharyngeal curve (pink) in B. Liquid gastric emptying is shown in C with a single exponential curve fitted to the time-activity curve for the stomach. The frequency of reflux to the pharynx/laryngopharynx is shown in D

Scintigraphic findings. All 7 patients showed evidence of significant intermittent or continuous full-column GER (Figure 1) with pharyngeal/ laryngopharyngeal contamination by refluxate. The average amplitude of the refluxate was 4.8 times higher than background and the average frequency of reflux to the larygopharynx in supine position was 22 episodes in 30 minutes (range=10-50 episodes). Liquid gastric emptying was normal in 2 patients (half-clearance time <16.0 minutes) while 5 were abnormal with half-clearance times ranging from 18.0 to 124.0 minutes (mean=56.8 minutes). Sample analysis is shown in Figure 1.

Analysis of the pattern of time-activity curves for the pharynx/laryngopharynx showed 5 rising curves when upright with 2 showing a falling pattern. When supine, 4 showed rising curves and 3 falling curves.

**Figure 2:**
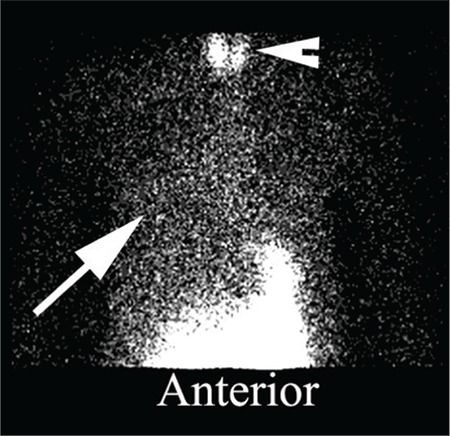
Delayed image of the anterior thorax obtained at two hours demonstrating aspiration of refluxate into predominantly the right lung (arrow). Radiopharmaceutical breakdown in the delayed image sometimes leads to free pertechnetate formation, which is taken up by the thyroid gland and is apparent in this study (arrowhead)

Six of the 7 cases showed evidence of pulmonary aspiration of refluxate in the delayed study (Figure 2) and the patient who did not show had rising time-activity curves for thelaryngopharyngeal region in the upright and supine positions.

**Figure 3:**
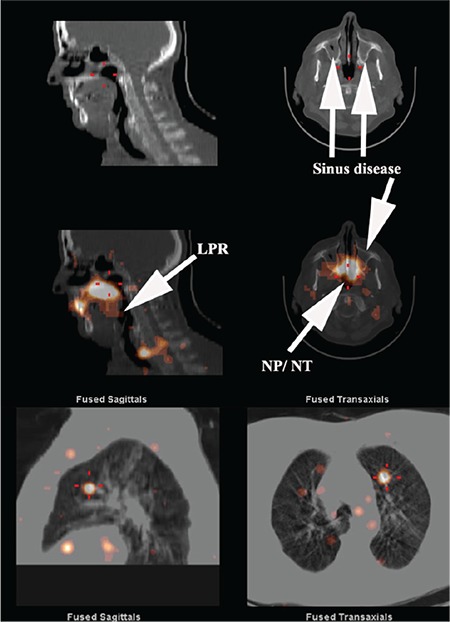
SPECT/CT image of head, neck and lungs. The CT image shows evidence of soft tissue thickening within the maxillary sinuses (arrows) consistent with sinus disease. The fused image in the middle panel demonstrates LPR, NP, NT and maxillary sinus contamination by refluxate. The lower panel shows aspirated refluxate within the lung tissue. Some misregistration is inevitable in the lungs due to respiratory motion and frequent coughing in many of these patients. SPECT: Single photon emission computed tomography, CT: Computed tomography, LPR: Laryngopharyngeal, NP: Nasopharyngeal, NT: Nasal turbinate

**Figure 4:**
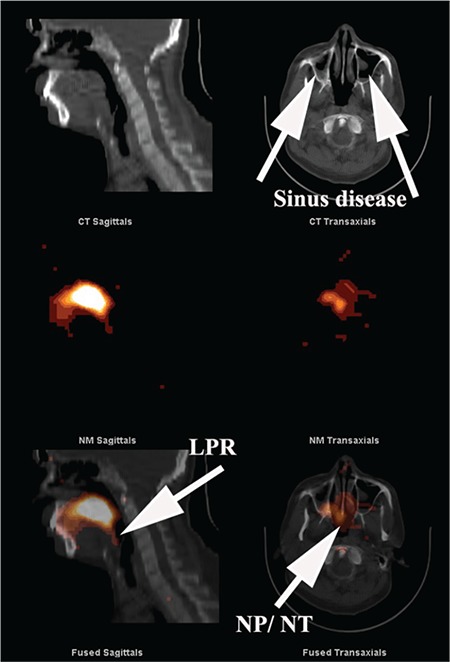
SPECT/CT image of head and neck. The upper panel of CT images with evidence of polyposis of both maxillary sinuses consistent with sinus disease. The fused images in the lower panel demonstrate evidence of LPR as well as contamination of the NP, NT and both maxillary sinuses. The central panel shows the scintigraphic images from the region in the absence of anatomical landmarks SPECT: Single photon emission computed tomography, CT: Computed tomography, LPR: Laryngopharyngeal, NP: Nasopharyngeal, NT: Nasal turbinate

**Figure 5:**
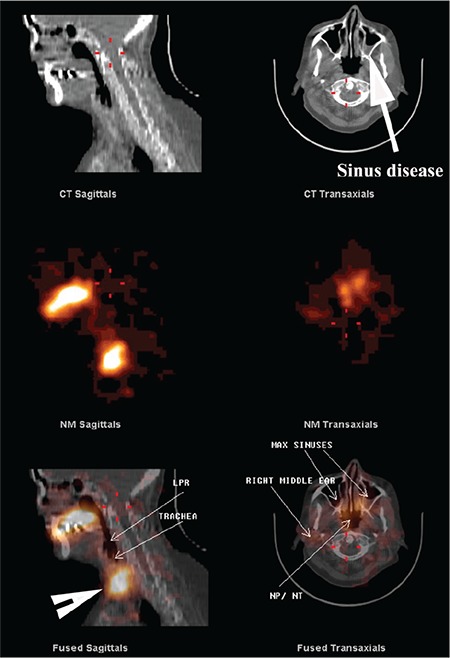
SPECT/CT image of head and neck. The upper panel of CT images demonstrates evidence of sinus disease in the left maxillary sinus. Fused images in the lower panel confirm the presence of refluxate contaminating the LPR, trachea, maxillary sinuses, NP, nasal NT and the right middle ear. The middle panel demonstrates the scintigraphic images and the difficulty in ascribing anatomical sites to these images without the CT study. Note uptake in the thyroid gland (arrowhead) in the fused sagittal image due to breakdown of the radiopharmaceutical in the delayed images SPECT: Single photon emission computed tomography, CT: Computed tomography, LPR: Laryngopharyngeal, NP: Nasopharyngeal, NT: Nasal turbinate

SPECT/CT imaging of the head, neck and lungs (Figure 3-4-5) demonstrated laryngopharyngeal contamination by refluxate in all 7, nasopharyngeal in 6 and maxillary sinus contamination in 5 patients. One patient had right middle ear contamination by refluxate. Lung aspiration of refluxate was confirmed in 6 patients.

## Discussion

This series raises a number of troubling issues pertaining to the relationship between rhinosinusitis and GERD. What is the role of GERD in sustaining the inflammatory process in the paranasal sinuses? Is it a primary cause or a promoter or is it a bystander phenomenon? How can one establish whether GERD involves the paranasal sinuses? What is the relationship between chronic fungal sinusitis and recurrent fungal pneumonia? Five of seven patients in this series were diagnosed as having asthma and treated for asthma although lung functions tests showed no reversibility after bronhcodilators. Many of these troubling issues can be explained by the scintigraphic reflux studies as both local disease in the oesophagus and extra-oesophageal structures can be physically visualised and at least semi-quantitated as shown. All 7 patients showed laryngopharyngeal contamination by refluxate with 6 also demonstrating lung aspiration. Laryngopharyngeal reflux was supported by the Belafsky scores of~20.0.

The majority of the patients in this case series gave a good history of established rhinosinusitis (8) predating the episodes of fungal pneumonia. They also had established and treated gastroesophageal reflux. It raises the question of how fungal infection can establish itself in the paranasal sinuses in an immune competent host. The combination of pseudostratified ciliated epithelium which is held together by tight junctions and protein secretions that have antimicrobial properties generally protects the host from infection (9). Breakdown of this barrier occurs in response to either allergic or inflammatory stimuli with secretion of epithelial-derived thymic stromal lymphopoietin, which in an up-regulated state may damage tight junctions, together with other toxic molecules such as the interleukins and interferon (9,10). All seven patients in this study had established GERD with the scintigraphic study showing paranasal sinus contamination by refluxate in the majority. There is a body of evidence that shows pepsin in refluxate as an agent capable of damaging nasal epithelium, which may have contributed or potentiated chronic rhinosinusitis (11). The seminal question is whether fungi initiate the inflammatory change or exploit a pre-existing inflammatory condition. The balance of opinion is that fungi initiate the inflammatory changes and then exploit the breakdown in defensive barriers (12). Luong et al. (13) demonstrated that common etiologic fungal antigens induced peripheral blood mononuclear cells to secrete elevated levels of interleukin 4 and 5 in patients with allergic fungal rhinosinusitis compared to normal controls. Inflammatory changes may also be promoted by coexistent staphylococcus aureus infection of the paranasal sinuses with the elevated IgE response to enterotoxin A and B, superantigens which are secreted by the bacterium (14). Pathogenic organisms such as fungi create a favourable environment for growth in the paranasal sinuses and subsequently produce conditions (eg. biofilms) that help evade the host immun system, antibiotic therapy and even surgical intervention (15).

The key finding that supports the hypothesis of recurrent infection of the lungs from established fungal disease of the paranasal sinuses is the response to laparoscopic fundoplication. It clearly implies a fundamental role for GERD with extra-oesophageal manifestations in the paranasal sinuses and subsequent aspiration of refluxate into the lungs. This is a complex issue as virtually all patients were on maintenance PPI therapy for the clinically diagnosed reflux disease. As has been shown, patients on high-dose PPI therapy, will continue to experience non-acid or even alkaline reflux which may be asymptomatic (16). This raises the possibility that PPI therapy in this cohort may have reduced the sterilisation effect of acid reflux on fungal disease in the paranasal sinuses and encouraged growth of the fungus in an alkaline environment. The change in pH of growth medium has been shown to induce fungal gene expression involved in the regulation of extracellular enzymes that may promote growth (17). While Aspergillus species are capable of growth across the entire range of pH from 2 to 11, they have been shown to be more tolerant to alkaline pH for growth (18). Furthermore, the alkaline pH with co-existent gastric mucositis may have reduced the absorption of many of the anti-fungal antibiotics utilised in these patients, with the exception of fluconazole (19).

GERD could play the role of transporter, particularly in its extra-oesophageal reach through the paranasal sinuses, laryngopharynx and airways as happened in all 7 patients. This system would need to be operational for a significant period, particularly when the patient was supine and the protective mechanisms against reflux were minimised, most likely during sleep (20,21). The possibility of assessing extra-oesophageal manifestations of GERD has until recently been a matter of deductive reasoning or based on observation of inflammatory change in the laryngopharynx on laryngoscopy (22). More recently, 24-hour impedance/pH monitoring has shown some promise although reproducibility has been an issue (23). The scintigraphic reflux technique utilised in the current study allows direct visualisation of refluxate within the sinuses, laryngopharynx and lungs (Figure 3, 4, 5) in addition to demonstrating disease within the oesophagus. Entry of refluxate into the paranasal sinuses was evident in 5 of 7 patients and aspiration into the lungs in 6 of 7 with recurrent fungal pneumonia. The scintigraphic reflux study assesses patients for lung aspiration after a sampling period of 2 hours, during which the patient is supine for only 30 minutes. Previous work has shown that rising time activity curves for the pharynx/laryngopharynx and upper oesophagus have a positive predictive value of 90% for lung aspiration of refluxate (5). The only patient who did not show aspiration in the 2-hour study had this pattern of activity for the laryngopharynx and upper oesophagus. It does suggest a high likelihood of aspiration during prolonged recumbency, especially during sleep (20,21).

The inability to eradicate fungal disease from the lungs has suggested more complex pathology, as even pulmonary lobectomy of the infected sites has not solved the problem. There has appeared to be a source of recurrent fungal infection, raising the strong possibility that the disease in the paranasal sinuses may have been the source, and the passage of refluxate through the sinuses, the main transport mechanism for infected tissue into the lungs. The only method of breaking the cycle of infection and re-infection has appeared to be to disrupt recurrent gastro-oesophageal reflux with surgical fundoplication. This technique effectively reduces the volume of reflux and interrupts what has appeared to be a recurrent transport system from the sinuses into the lungs in this particular group of patients.

Diagnosis of aspergillosis within the paranasal sinuses has proven to be problematic as engendering fungal growth in external media has sometimes been unproductive. However, microscopy of biopsies from the sinuses with silver staining or molecular techniques has demonstrated semi-invasive disease of the mucosa with a diagnosis of allergic fungal sinusitis with polyposis. This is important, as studies of normal (uninfected) paranasal sinuses has shown a biome in which fungal elements may be inhaled as airborne contaminants and trapped in the paranasal mucous, being shuttled to the oropharynx for removal (24). One of the significant problems with molecular techniques is the detection of molecular material of inactive microorganisms (24). The inability to culture fungi from biopsy material may be related to the formation of biofilms, which inhibits growth in culture media, as has been shown in a number of reviewed studies (24). The type of biofilm (eg. haemophilus influenza versus staphylococcus aureus) may affect the severity of disease by protecting pathogenic organisms from the effects of antibiotics in chronic rhinosinusitis and even affect the success of surgery (15,25,26).

## Conclusion

Fungal pneumonia in the immunocompetent host is rare. It may well begin in the paranasal sinuses, as has been shown in one of the larger series and when coupled with the appropriate pre-conditions become chronic and increase the risk of spread to the lungs. This series offers a good case to support the hypothesis that recurrent fungal pneumonia may be due to re-infection of the lungs from allergic fungal sinusitis with polyposis by passage of GER through the paranasal sinuses and into the lungs.
